# The impact of women’s empowerment on their children’s early development in 26 African countries

**DOI:** 10.7189/jogh.10.020406

**Published:** 2020-12

**Authors:** Fernanda Ewerling, John W Lynch, Murthy Mittinty, Anita Raj, Cesar G Victora, Carolina VN Coll, Aluisio JD Barros

**Affiliations:** 1 International Center for Equity in Health, Federal University of Pelotas, Pelotas, Brazil; 2 Postgraduate Program in Epidemiology, Federal University of Pelotas, Pelotas, Brazil; 3 School of Public Health, University of Adelaide, Adelaide, SA, Australia; 4 Population Health Sciences, Bristol Medical School, University of Bristol, Bristol, UK; 5 Center on Gender Equity and Health, University of California San Diego, San Diego, California, USA

## Abstract

**Background:**

Every year more than 200 million children under-five years fail to achieve their full developmental potential in low- and middle-income countries (LMICs). Although women´s empowerment has been associated with improved child health and development outcomes, this is a topic little studied in LMICs. We investigated the associations between women´s empowerment and early childhood development among a sample population of 84537 children aged 36-59 months from national health surveys of 26 African countries.

**Methods:**

We used data from Demographic and Health Surveys (DHS) and Multiple Indicator Cluster Surveys (MICS) ranging from 2010 to 2018. Four developmental domains were assessed among children using the Early Childhood Development Index: literacy-numeracy, physical, learning and socioemotional. Women’s empowerment in attitude to violence, social independence and decision-making was evaluated using the SWPER global, a validated survey-based index. We reported effect sizes for each country and a combined estimate of the association. The study covers all countries with surveys in the region and uses a novel approach for measuring women’s empowerment, the SWPER.

**Results:**

Across all countries, 15.1% of the children were on track in the literacy-numeracy domain, 92.3% in physical, 81.3% in learning and 67.8% in socio-emotional. The odds of a child being on track in literacy-numeracy increased by 34% (odds ratio (OR) = 1.34; 95% confidence interval (CI) = 1.31-1.37), 88% (OR = 1.88; 95% CI = 1.85-1.91) and 34% (OR = 1.34; 95% CI = 1.29-1.39), with a one standard deviation increase in the scores of attitudes to violence, social independence and decision-making domains of empowerment, respectively. No effect of empowerment was observed for the other domains of child development.

**Conclusions:**

Our results show a consistent positive effect of empowerment on the literacy-numeracy domain of child development cross-nationally in Africa and this was particularly evident for the social independence domain of the SWPER. Programs and interventions may also consider addressing the reduction of gender inequalities to improve child development.

Every year more than 200 million children under-five years of age from low- and middle-income countries (LMICs) fail to achieve their full potential in cognitive development, most in south Asia and sub-Saharan Africa [1]. In 2015, the international community embraced this cause, including universal access to early childhood development (ECD), care and preprimary education as a target on the Sustainable Development Goals (SDGs). This signals an important change relative to the earlier Millennium Development Goals, which only targeted child survival. International organizations, governments and policy makers are now committed to guarantee that all children are able to not only survive, but also thrive [2]. The first five years of the child’s life are critical for their cognitive, social, and physical development [3]. This period is an important window for investments, since interventions focused on ECD are among the most cost-effective approaches to increase educational achievements and productivity in adulthood.[2,4] Failing to promote child development may also sustain the intergenerational transmission of poverty [1,5].

The family environment is a key determinant of child survival and optimal development [5], which comprises, among other factors, a stimulating environment, social interactions with dedicated caregivers and adequate nutrient intake [4,6]. Poverty, discrimination, conflict and other forms of individual, family and community stress create barriers for families to provide nurturing care for children and may compromise their ability to parent effectively [7]. This may be exacerbated in contexts where gender inequalities and women’s marginalization are common [8,9]. In LMICs, many women are still deprived of claiming their rights and of being able to make decisions about the direction of their lives and those of their children [10,11]. With potential to promote economic growth, reduction of poverty and being an important accomplishment of human rights, the empowerment of women is also targeted by the SDGs [12]. More empowered women are more likely to use modern contraceptives and to have access to antenatal care and skilled birth attendance [13-17]. They also generally desire a smaller number of children, which should allow them to provide better care to each child [18,19]. Data on ECD are still limited in LMICs. UNICEF recently started collecting data on the Early Childhood Development Index (ECDI), which allows the assessment of four developmental domains: literacy-numeracy, physical, socioemotional and learning. To our knowledge, no study has ever evaluated the association between the mothers’ empowerment level and ECD in LMICs. This study adds to the literature by analyzing this association in African countries, which may present high gender inequalities and low levels of child development [6,20].

## METHODS

We used data from Multiple Indicator Cluster Surveys (MICS) and Demographic and Health Surveys (DHS) as both have similar questionnaires and sampling strategies. Items to assess ECD have been added to the MICS questionnaires since 2009, and in some DHS since 2010 [21]. We selected the latest survey for each African country for which the ECD module had been applied, either in MICS or DHS. A total of 26 countries were included (see **Table 1**).

**Table 1:** Sample sizes and prevalence (%) of children aged 36-59 months who are developmentally on track in literacy-numeracy, physical, learning and social-emotional domains, and in the Early Child Development Index

				% children 36-59 months who are on track by developmental domain*					
**Region**	**Country**	**Year**	**Source**	**Literacy- numeracy**	**Physical**	**Learning**	**Social- emotional**	**ECDI†**	**Sample size‡**
Eastern & Southern Africa	Burundi	2016	DHS	9.1	92.6	63.7	59.2	41.1	4254
	Eswatini	2014	MICS	20.6	93.7	93.6	64.9	66.8	512
	Malawi	2013	MICS	17.6	89.3	80.5	72.0	60.8	5893
	Rwanda	2014	DHS	7.3	95.2	86.2	81.9	71.3	2240
	Uganda	2016	DHS	29.1	90.9	86.8	67.3	64.7	4129
	Zimbabwe	2014	MICS	9.9	92.4	87.1	65.1	59.9	2813
Middle East & North Africa	Algeria	2012	MICS	28.2	96.1	89.3	70.3	69.9	5033
	Tunisia	2018	MICS	39.9	95.7	91.6	81.4	82.8	1384
West & Central Africa	Benin	2017	DHS	8.7	87.4	71.9	68.9	53.8	4170
	Central African Republic	2010	MICS	7.1	95.5	74.1	58.1	46.6	2905
	Cameroon	2011	DHS	17.2	92.8	82.5	52.4	49.8	1641
	Chad	2014	DHS	5.3	83.5	54.0	59.5	32.1	3826
	Congo Brazzaville	2011	DHS	12.8	87.5	80.3	55.5	49.1	1968
	Congo DR	2013	DHS	10.4	92.2	79.9	78.3	64.5	2458
	Cote d’Ivoire	2016	MICS	6.9	95.0	87.0	70.0	63.5	2677
	Gambia	2018	MICS	13.3	94.0	93.5	66.3	65.2	3500
	Ghana	2011	MICS	29.3	97.4	89.4	74.3	75.1	2329
	Guinea	2016	MICS	6.6	92.2	78.8	62.0	48.8	2530
	Guinea-Bissau	2014	MICS	7.2	89.8	86.8	72.7	61.1	2081
	Mali	2015	MICS	7.9	94.4	84.6	73.1	61.9	5601
	Mauritania	2015	MICS	26.1	90.7	79.1	66.4	59.6	3367
	Nigeria	2016	MICS	28.2	89.8	77.5	71.2	60.7	9699
	São Tome & Principe	2014	MICS	16.9	94.0	78.5	61.0	53.8	568
	Senegal	2017	DHS	4.4	95.3	86.3	75.0	66.5	3618
	Sierra Leone	2017	MICS	14.3	89.8	79.7	59.6	51.2	3318
	Togo	2013	DHS	8.4	92.6	70.4	75.4	53.9	2053
**Overall**				**15.1**	**92.3**	**81.3**	**67.8**	**59.0**	**84537**

ECDI – Early Child Development Index, DHS – Demographic and Health Surveys, MICS – Multiple Indicator Cluster Surveys

*Please see **Box 1** for the items included in each developmental domain.

†Children are considered developmentally on track on the Early Child Development Index (ECDI) if they are on track in at least three of the four developmental domains.

‡Children aged 36-59 months living with the mother; restricted to partnered mothers.

### Early child development

Early child development was measured by the ECDI developed by UNICEF intended for cross-cultural comparisons. This is a multidimensional index composed of ten questions directed to the child’s mother or primary caregiver designed to assess the development of children aged 36 to 59 months [4]. The ECDI covers four developmental domains: literacy-numeracy, physical, learning and social-emotional. The response categories for all questions are *“yes”*, *“no”*, and *“don’t know”*. For each domain, the child is considered on track according to the number of items with affirmative answers. For example, the literacy-numeracy domain is composed of three items (child can identify/name at least ten letters of the alphabet; child can read at least four simple, popular words; and child knows the name and recognizes the symbol of all numbers from 1 to 10). The child is considered on track in this domain if at least two of these three items are achieved. Overall, the child is considered developmentally on track if at least three of the four domains were considered on track. The complete description of the ECDI questions and its operationalization to determine whether the child is developmentally on track in each domain is presented in **Box 1**. We analyzed each ECDI domain separately and the composite ECDI score according to this operationalization.

Box 1 **Operationalization of the Early Child Development Index (ECDI) in children aged 36-59 months.**

(1) *Literacy-numeracy*: Children are considered developmentally on track if at least two of the following are true: Can identify/name at least ten letters of the alphabet; can read at least four simple, popular words; knows the name and recognizes the symbol of all numbers from 1 to 10.

(2) *Physical:* Children are considered developmentally on track if one or both of the following is true: can pick up a small object with two fingers, like a stick or a rock from the ground; is **not** sometimes too sick to play.

(3) *Learning:* The child is identified as being developmentally on track if one or both of the following is true: follows simple directions on how to do something correctly; when given something to do, is able to do it independently.

(4) *Social-emotional:* The child is identified as being developmentally on track if at least two of the following are true: gets along well with other children; does **not** kick, bite, or hit other children; does **not** get distracted easily.

Overall, the child is developmentally on track if at least three of the four component domains were considered to be on track [4].

### Women’s empowerment

Mother´s empowerment was measured by the Survey-based Women’s emPowERment (SWPER) global index [22]. This is an individual level measure which assesses three domains of empowerment: attitude to violence, social independence and decision making. It is based on 14 questions related to the women’s responses on whether beating the wife is justified in some situations, to who makes decisions in the household (regarding the respondent’s health care, large expenses and visits to family and relatives), access to information, educational attainment, age at marriage and first child, and differences in age and education relative to the husband (Table S1 in the **Online Supplementary Document**). The SWPER global provides continuous standardized scores, so that a zero score means that the woman is at an average level of empowerment compared to the set of low- and middle-income countries used to develop the revised version of the index. A positive score indicates higher empowerment than average and a negative score, the opposite. Full details on the construction of the index and its validity are presented elsewhere [22,23].

### Missing data

Some women did not have complete information on all the items needed to calculate the SWPER global scores and some surveys included in this analysis did not collect information on some variables required for calculating the SWPER global. For instance, MICS does not provide the husband’s education nor any information on the woman’s participation in household decisions. To overcome the husband’s education issue, we used the schooling of the head of the household as a proxy for the husband’s education, and other missing variables were imputed. We used multiple imputation for these missing variables by design (questions not included in the survey questionnaire) and for those cases where the answers for individual women were missing. We assumed that the data were missing at random. We pooled the data from the 26 countries used in this analysis with data from the latest DHS conducted in each African country since 2010 with available information on all SWPER variables (these surveys collected information on the SWPER items but not on child development). By doing so the imputation model relied on a much larger sample. Thus, multiple imputation was performed in this data set that contained information on 493 764 partnered women from 41 African countries. In the pooled data set, 38% of the women (n = 187 521) had missing information for at least one SWPER item (varying from 100% in MICS, that do not collect any information on decision-making, to 8.9% in DHS surveys). The literature advocates that five imputed data sets are generally sufficient, but up to 20 data sets are preferable to deal with the sampling variability from the imputation process. Given the considerably high proportion of missing cases in our data, we decided to impute 20 data sets. The SWPER items and the outcome of interest (ECDI) with missing information were imputed based on country, wealth index, and area (urban/rural) of residence, woman’s age and the outcome of interest (ECDI). Women in the attitude to violence items women could answer “yes”, “no” or “don’t know” (see Table S1 in the **Online Supplementary Document**). The latter is also considered as an information in the SWPER, but the proportion of women answering “don’t know” in these items is too low (generally <1%), causing the multiple imputation not to converge. Thus, we set this information to missing and the imputation process also imputed information (yes/no) for these items. The distribution of the empowerment scores with imputed items was very similar to the distribution of the complete cases (Figure S1 in the **Online Supplementary Document**). For the MICS surveys, the decision-making items were imputed to allow the calculation of the attitude to violence and social independence domains, but the decision-making domain are only analyzed in the DHS.

### Statistical analyses

We included in the analyses children aged 36-59 months living with the mother at the time of the interview. The ECDI module is only applied for the youngest child in the age range in DHS surveys, so the analyses were also restricted to similar children in MICS to allow comparison. As most of the questions related to women’s empowerment available in the surveys were restricted to partnered women, the SWPER global index can only be calculated for this group. Thus, children whose mothers did not have a partner at the time of the survey were not included in the analysis. The proportion of children developmentally on track in each developmental domain using all these restrictions was very similar to the results found considering all children assessed in the surveys (results not shown). Most differences were smaller than 1 percentage point (either positive or negative), with an average of 0.0 percentage points for the literacy-numeracy and social-emotional domains and -0.1 for physical and learning.

We used logistic regression to assess the association between maternal empowerment and child development according to the composite ECDI index as well as each ECDI domain. Crude and adjusted analyses were performed to evaluate whether adjusting for household wealth would affect the results. Preliminary analyses suggested no clear evidence that the sex of the child modified the associations, thus all children were analysed together. Rubin’s rules were applied to combine the results of the multiple imputed data sets utilizing the command ‘*mi estimate*’ on the statistical software Stata (StataCorp. Stata Statistical Software: release 14. College Station, TX: StataCorp LP; 2015). To get an overall effect and achieve higher statistical power, meta-regressions across countries were performed to assess the pooled odds ratios for the association between empowerment and the child development domains. Meta-regressions combine the results of multiple studies (or, in this case, of multiple countries), weighting each study result by their sample size. Lastly, we performed sensitivity analysis by generating a new social independence measure that excluded the mothers’ education and executed the same logistic and meta-regressions to check whether the observed associations would persist. The command ‘*svy’* was used in all the analyses to account for the surveys’ sample design. Both DHS and MICS are public sources of information and ethical approval was obtained from the national institutions involved in each survey.

## RESULTS

Survey data collection ranged from 2010 to 2018, with 18 surveys from West and Central Africa, two from Middle East and North Africa and six from Eastern and Southern Africa (**Table 1**). In twelve countries (Burundi, Rwanda, Zimbabwe, Benin, Central African Republic, Chad, Cote d’Ivoire, Guinea, Guinea-Bissau, Mali, Senegal and Togo), less than 10% of the children were on track on literacy-numeracy. Ghana and Tunisia presented the best performance in this domain, with 29.3% and 39.9% of the children being on track, respectively. The average proportion across all countries was 15.1%. A vast majority of children were considered on track for the physical and learning developmental domains (92.3% and 81.3%, respectively). The physical domain showed the smallest variability across countries, ranging from 83.5% in Chad to 97.4% in Ghana. Regarding the social-emotional domain, 67.8% of the children were on track, on average. Overall, 59.0% of the children were on track for the composite ECDI (ie, on track in at least three out of the four domains). Chad presented the worst performance, with only 32.1% of the children on track on the composite ECDI, contrasting with Tunisia where more than 80% of children were on track (**Table 1**).

**Figure 1:**
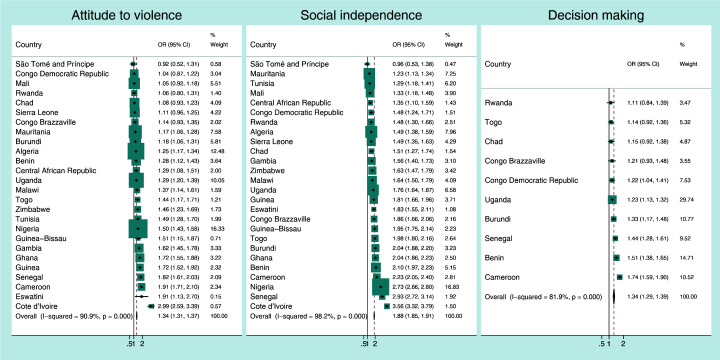
Association between *literacy-numeracy development* of the child and the mothers’ empowerment level for each SWPER global domain. Coefficients are the crude odds ratios (OR) for a standard deviation increase in the SWPER global score.

**Figure 2:**
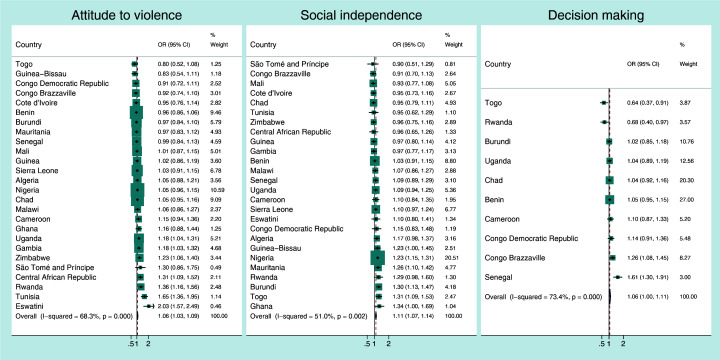
Association between *physical development* of the child and the mothers’ empowerment level for each SWPER global domain. Coefficients are the crude odds ratios (OR) for a standard deviation increase in the SWPER global score.

**Figure 3:**
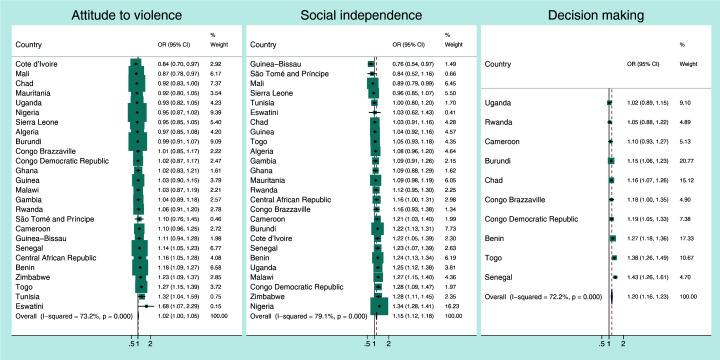
Association between *learning development* of the child and the mothers’ empowerment level for each SWPER global domain. Coefficients are the crude odds ratios (OR) for a standard deviation increase in the SWPER global score.

**Figure 4:**
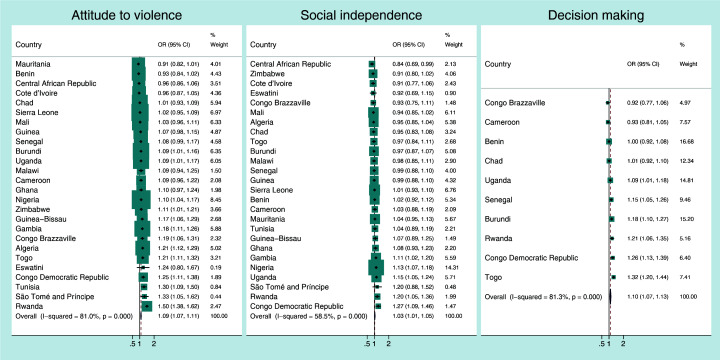
Association between *socio-emotional development* of the child and the mothers’ empowerment level for each SWPER global domain. Coefficients are the crude odds ratios (OR) for a standard deviation increase in the SWPER global score.

**Figure 1**, **Figure 2**, **Figure 3**, **Figure 4** present the crude associations between the mothers’ empowerment level as measured by the three domains of the SWPER global (attitude to violence, social independence and decision-making) and each child developmental domain separately. The effect measures can be interpreted as odds ratios for a standard deviation increase in the SWPER global score. The figures also present the overall associations for all the countries, weighted by their sample sizes.

There was substantial heterogeneity in the effects of empowerment on child development among countries. For attitude to violence the associations with literacy-numeracy development tended to be positive, but in seven out of 26 countries the confidence intervals included the unity (**Figure 1**). All countries presented positive associations between mother’s social independence and literacy-numeracy scores, except for São Tome and Principe there was no association (OR = 0.93; 95% CI = 0.53-1.38). In Cote d’Ivoire one standard deviation in the mother’s attitude to violence or social independence increased the odds of the child being on track in literacy-numeracy by 3-fold (OR = 2.99; 95% CI = 2.59-3.39 for attitude to violence and OR = 3.56; 95% CI = 3.32-3.79 for social independence). Fewer countries had information on the decision-making score, but associations also tended to be positive. Overall, for each standard deviation increase in attitude to violence, social independence and decision-making scores, the pooled odds for the child being on track on literacy-numeracy increased 34% (OR = 1.34; 95% CI = 1.31-1.37 I^2^ = 90.9%), 88% (OR = 1.88; 95% CI = 1.85-1.91; I^2^ = 98.2%) and 34% (OR = 1.34; 95% CI = 1.29-1.39; I^2^ = 81.9%), respectively. We performed sensitivity analyses by excluding education from the social independence domain, as it could be driving the positive results. However, the overall association was not strongly affected by this change (pooled OR = 1.72; 95% CI = 1.69-1.75).

Generally, the associations between the three empowerment domains and the physical, learning and social-emotional development of the child were small (**Figure 2**, **Figure 3**, **Figure 4**). The pooled odds ratios for physical development were 1.06 (95% CI = 1.03-1.09; I^2^ = 68.3%), 1.11 (95% CI = 1.07-1.14; I^2^ = 51%) and 1.06 (95% CI = 1.00-1.11; I^2^ = 73.4%), respectively, for attitude to violence, social independence and decision-making domains.

We repeated the analyses after adjusting for household wealth. These results can be found in Figures S2-S5 in the **Online Supplementary Document****.** Generally, the associations between empowerment and the literacy-numeracy scores were markedly attenuated, but the patterns remain similar to the crude analyses. After such adjustment, each additional standard deviation in attitude to violence, social independence and decision-making increased the pooled odds of being on track in literacy-numeracy by 11% (OR = 1.11; 95% CI = 1.08-1.14), 34% (OR = 1.34; 95% CI = 1.31-1.37) and 18% (OR = 1.18; 95% CI = 1.13-1.23), respectively. In the other domains, with a few exceptions, the overall associations were very small or null after adjustment by wealth. The combined associations of attitude to violence and social independence on the physical development of the child after adjustment were reduced to 3% (OR = 1.03; 95% CI = 1.00-1.06) and 5% (OR = 1.05; 95% CI = 1.01-1.08), respectively. A similar situation happened for the associations of attitude to violence and decision making on the social-emotional development of the child where the pooled odds were reduced to 8% (OR = 1.08; 95% CI = 1.06-1.10) and 9% (OR = 1.09; 95% CI = 1.06-1.12), respectively. For the learning development, social independence and decision-making domains, the odds of the child being on track increased by 4% (OR = 1.04; 95% CI 1.01-1.07) and 16% (OR = 1.16; 95% CI = 1.12-1.20) for each standard deviation increase in the mothers’ empowerment scores after adjustment.

We also analyzed associations with the composite ECDI score (Figure S6 in the **Online Supplementary Document**). Overall, the unadjusted pooled odds for the attitude to violence, social independence and decision-making domains were, respectively, of 11% (OR = 1.11; 95% CI = 1.09-1.13), 21% (OR = 1.21; 95% CI = 1.19-1.23) and 19% (OR = 1.19; 95% CI = 1.16-1.22) on the ECDI. With a few exceptions – Chad, Mali, Cote d´Ivoire, Mauritania and Sierra Leone in attitude to violence and Mali and Sao Tome and Principe in social independence – all countries presented positive associations. After adjustment for wealth, the overall odds were reduced to 5% (OR = 1.05; 95% CI = 1.03-1.07; I^2^ = 77.4%), 8% (OR = 1.08; 95% CI = 1.06-1.11; I^2^ = 64%) and 15% (OR = 1.15; 95% CI = 1.11-1.18; I^2^ = 61.9%) for the attitude to violence, social independence and decision-making domains, respectively (Figure S7 in the **Online Supplementary Document**).

## DISCUSSION

To our knowledge, no study to date has examined the associations between women’s empowerment and ECD in LMICs. On average, 15.1%, 92.3%, 81.3% and 67.8% of the children were on track for literacy-numeracy, physical, learning and socioemotional developmental domains, respectively. Despite substantial heterogeneity between countries in the association of mother’s empowerment with ECD, we showed that generally higher maternal empowerment levels – particularly for the social independence domain – were strongly associated with the acquisition of literacy-numeracy skills in several African countries. In contrast, the mothers’ empowerment level presented weak or null associations with the three other developmental domains.

In the African context, where many countries are still struggling to have reliable data on child health, obtaining valid and representative data on child development remains a major challenge [2,7]. The ECDI was developed by UNICEF in order to fill this gap, yet this indicator is not free of limitations. Almost all children were on track in the physical and learning domains, which suggests the presence of a ceiling effect for these measures. Generally, the items comprised in both domains are easily achieved, so that almost all children are considered as being on track. The weak associations between empowerment (as it would be expected with any other predictor) and these two domains may be due to the tests’ inability to detect children who present less marked degrees of impairment. For example, in the physical domain, the pincer grasp item represents a skill normally developed before one year of age, so it would only capture severe developmental setbacks in children aged 36-59 months [21] · The same authors argue that being too sick to play – also in the physical domain – would represent the children’s health status rather than their early developmental skills.

We found that each additional standard deviation in the social independence empowerment level of the mother was associated with an almost doubling in the child’s odds of being on track on literacy-numeracy. This empowerment domain is comprised by items related to the woman’s education, ages at first birth and at first cohabitation, and the differences in age and education between the woman and her partner. There is a large body of literature reporting on the effect of parental education on the development of the child [6,24,25]. However, our sensitivity analyses showed that this association was not solely driven by education, suggesting that it represents a possible effect of the social independence as a whole.

In spite of the limitations of the ECDI, the availability of a cross-culturally validated standard measure is an extremely valuable advance in the field. In many LMICs, this is the only child development measure available. The ECDI opens innumerable new research opportunities and has potential to make governments and policy makers accountable regarding the countries’ advances. However, the ceiling effects may indicate that the ECDI will require adjustments to allow the monitoring of the progress towards the 2030 SDGs.

Our study has limitations that should be considered while interpreting the findings. Female-headed households are generally more likely to face poverty and possibly poor health outcomes than those where the child’s father is also present [26], yet as it currently stands, the women’s empowerment measure can only be calculated for women who were in a union; in the surveys included in the analyses, the median proportion of female-headed households was 25.2% (range from 8.1% to 45.6%). Additionally, children who do not live with their mothers were not included in our analyses. Therefore, we cannot extrapolate the results to all children aged 36-59 months in the countries analyzed, but the ones included in our study are certainly the large majority. Also, the heterogeneity of the effects between countries was quite high, so the pooled effect estimated in the metanalyses should be interpreted with caution. Further studies are necessary to identify the possible reasons for the high heterogeneity.

Women’s empowerment is strongly associated with family socioeconomic characteristics [23,27]. This association is complex, because poverty may contribute to reduce empowerment, but on the other hand empowered women are more likely to work outside the home and contribute to household income [28]. ECD is also closely associated with family wealth [29]. By adjusting the analyses for household wealth, we aimed to evaluate whether the association between empowerment and child development persisted. After adjustment, the association was substantially attenuated – for example, the odds for literacy-numeracy according to social independence fell from 1.90 to 1.35. Other odds ratios also showed less marked attenuation, but the associations remained positive (results presented in Appendix S1 of the **Online Supplementary Document**). One should, however, be careful while interpreting these associations, given that the relationship between wealth and women´s empowerment is complex and possibly bidirectional. By adjusting the association by wealth, we attempt to account for it as a confounder. But it is also possible that wealth is part of the pathway linking empowerment to child development.

Empowering women is a goal in itself, but it also has a great potential to improve health and economic outcomes both at household and community level. We showed that maternal empowerment is associated with better ECD in the literacy-numeracy domain. Thus, programs and interventions aimed at improving the future human capital of today’s children may also consider addressing the empowerment of women and the reduction of gender inequalities.

## Additional material

Online Supplementary Document
