# Supplementary material for: The impact of women’s empowerment on their children’s early development in 26 African countries
Source: J Glob Health. 2020 Aug 14;10(2):020406. doi: 10.7189/jogh.10.020406 (PMC7649042; doi:10.7189/jogh.10.020406)

Table S1. Variables used in the development of the survey-based women's empowerment index.

SWPER Domain	Variable	Code or unit
Attitude to violence	Beating NOT justified if:	
	1. wife goes out without telling husband	Yes = -1;
	2. wife neglects the children	Don't know=0[2];
	3. wife argues with husband	No=1
	4. wife refuses to have sex with husband	
	5. wife burns the food	
Social independence	6. Frequency of reading newspaper or magazine	Not at all=0; <once a week=1; ≥once a week=2
	7. Woman education in completed years of schooling	Years
	8. Education difference: woman minus husband completed years of schooling	Years
	9. Age difference: woman age minus husband age	Years
	10. Age at first cohabitation	Years
	11. Age of woman at first birth	Years
Decision making	Who usually decides on:	
	12. Respondent's health care	Husband or other alone= -1;
	13. Large household purchases	Respondent alone/ jointly with husband or other=1
	14. Visits to family or relatives	

Note: Category "Don't know" was set to missing and imputed as yes or no in the multiple imputation process.

Figure S1. Comparison of the SWPER score distribution between complete cases[1] and observations with at least one SWPER item imputed using multiple imputation.

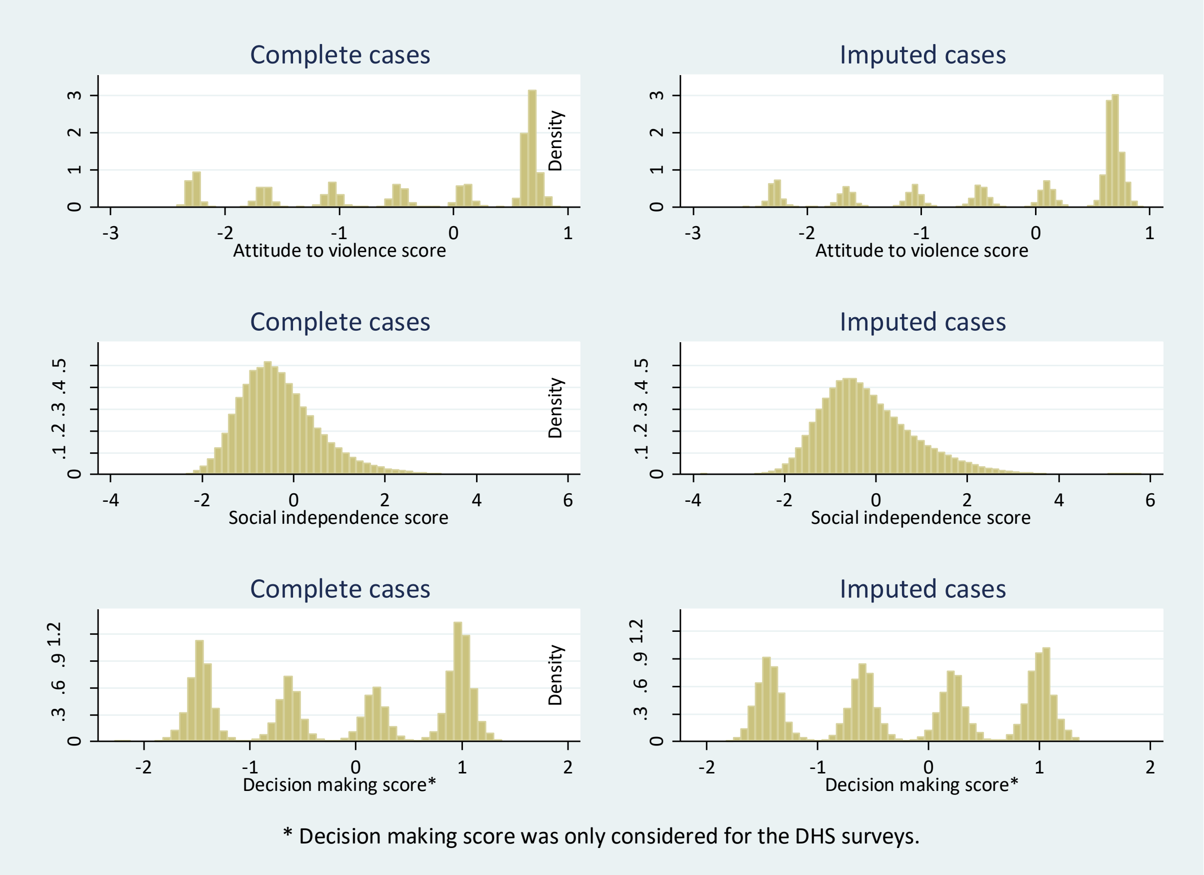

Note: Complete cases are all women with complete information on the 15 items that compose the SWPER Index; imputed cases are the ones with at least one missing item.

Figure S2. Association between *literacy-numeracy* development of the child and the mother's empowerment level for each SWPER global domain. Coefficients are the odds ratios (OR) for a standard deviation increase in the SWPER global score adjusted for wealth.

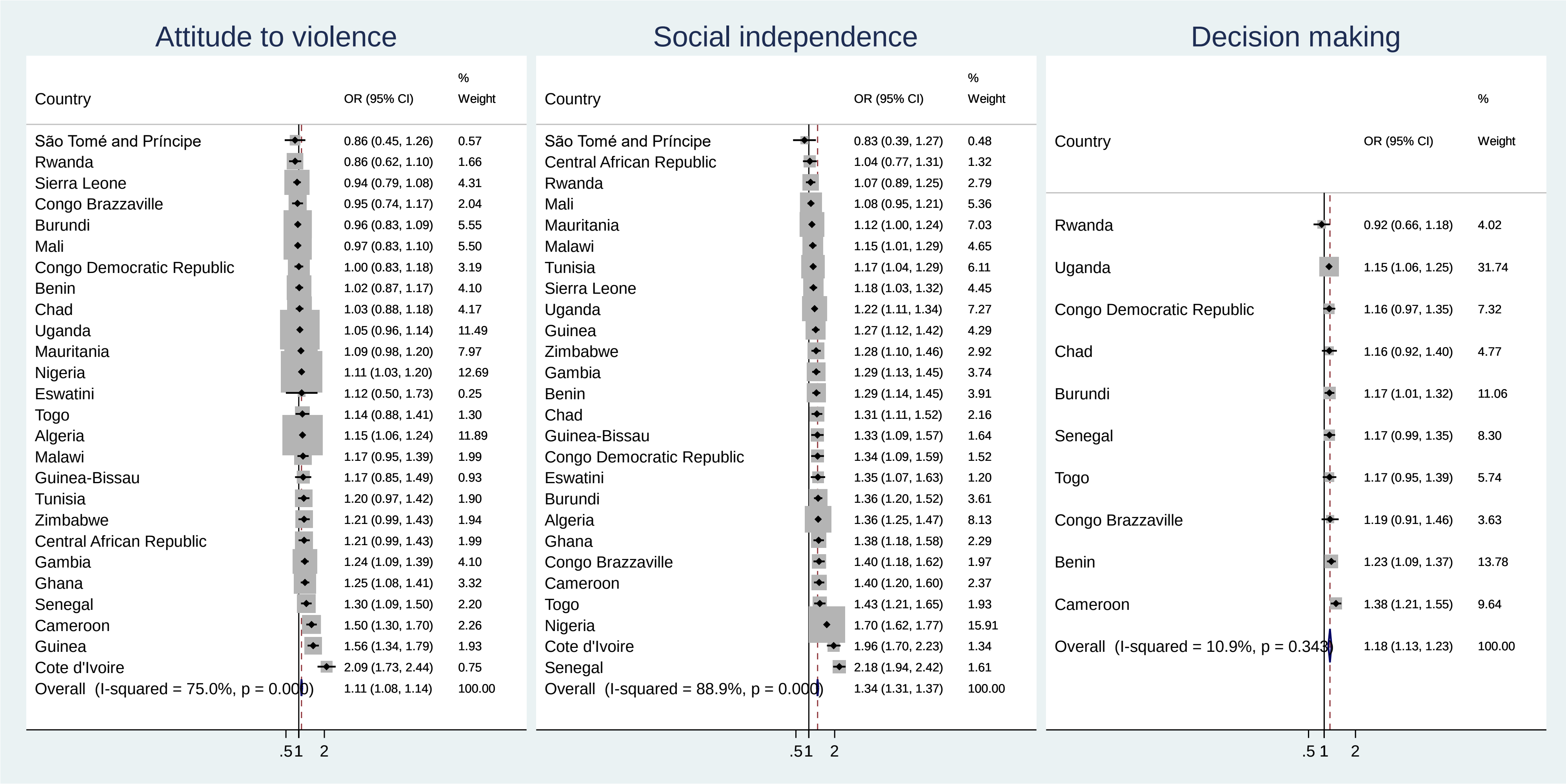

Figure S3. Association between *physical* development of the child and the mother's empowerment level for each SWPER global domain. Coefficients are the odds ratios (OR) for a standard deviation increase in the SWPER global score adjusted for wealth.

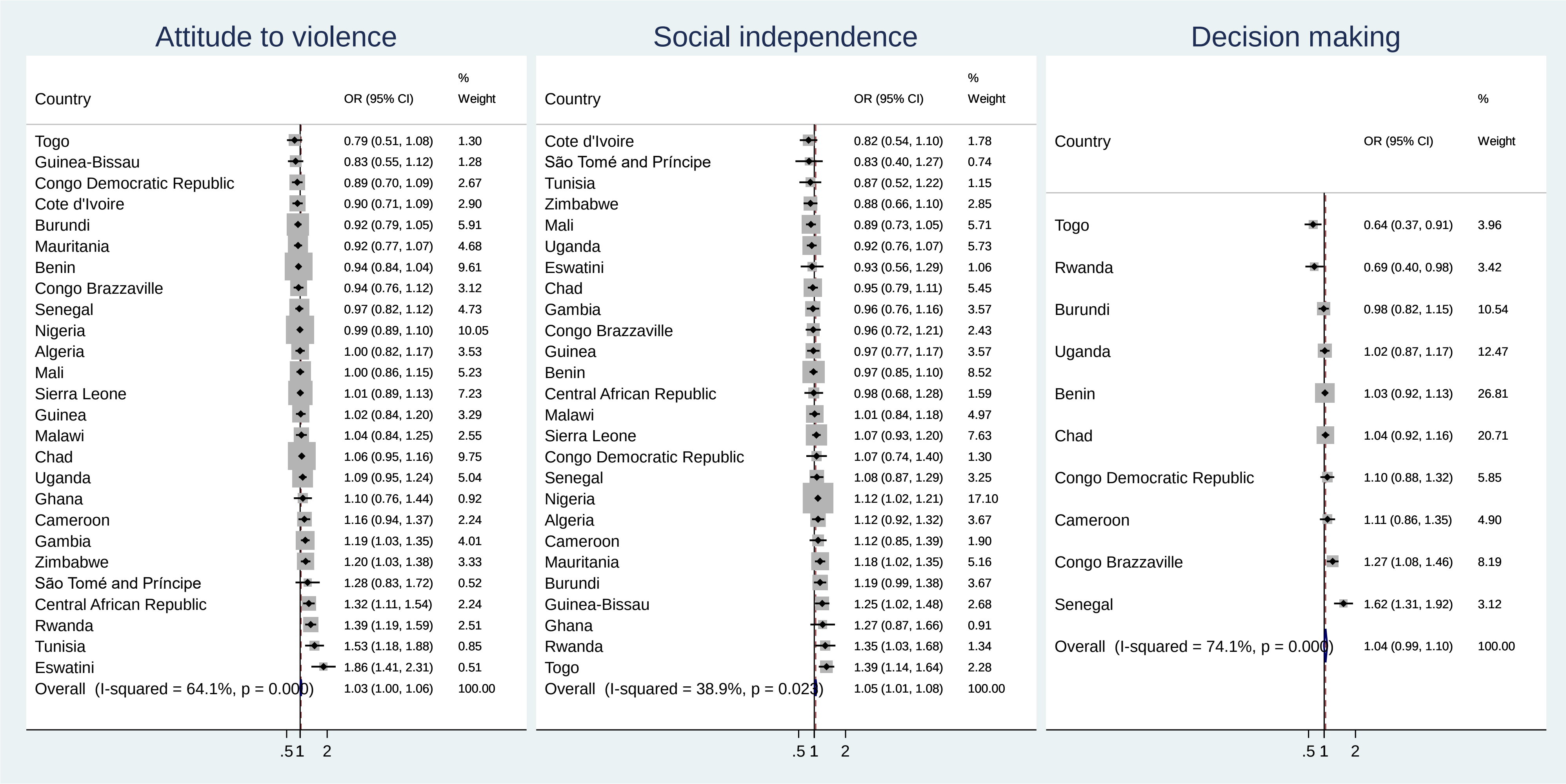

Figure S4. Association between *learning* development of the child and the mother's empowerment level for each SWPER global domain. Coefficients are the odds ratios (OR) for a standard deviation increase in the SWPER global score adjusted for wealth.

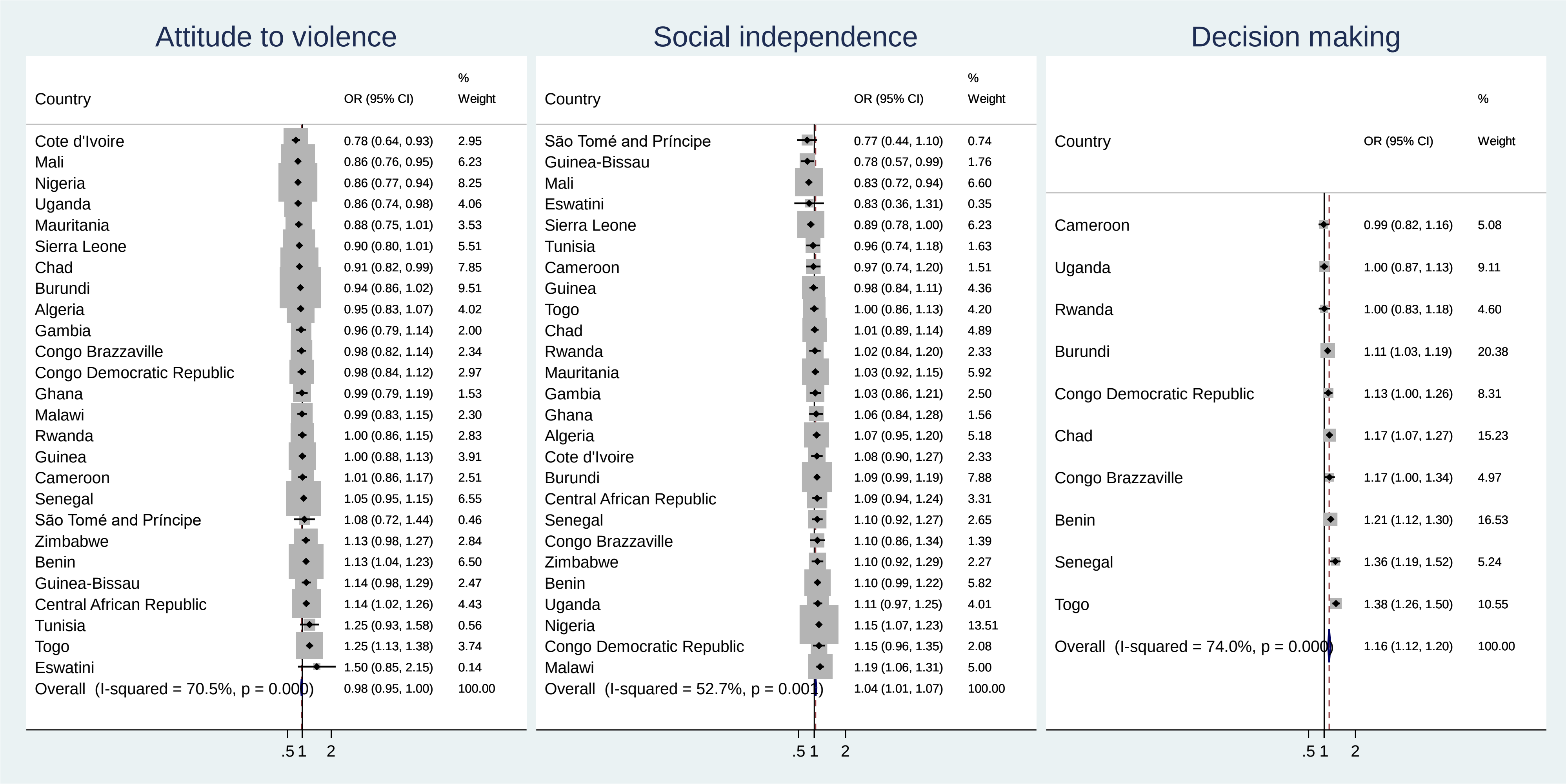

Figure S5. Association between *socio-emotional* development of the child and the mother's empowerment level for each SWPER global domain. Coefficients are the odds ratios (OR) for a standard deviation increase in the SWPER global score adjusted for wealth.

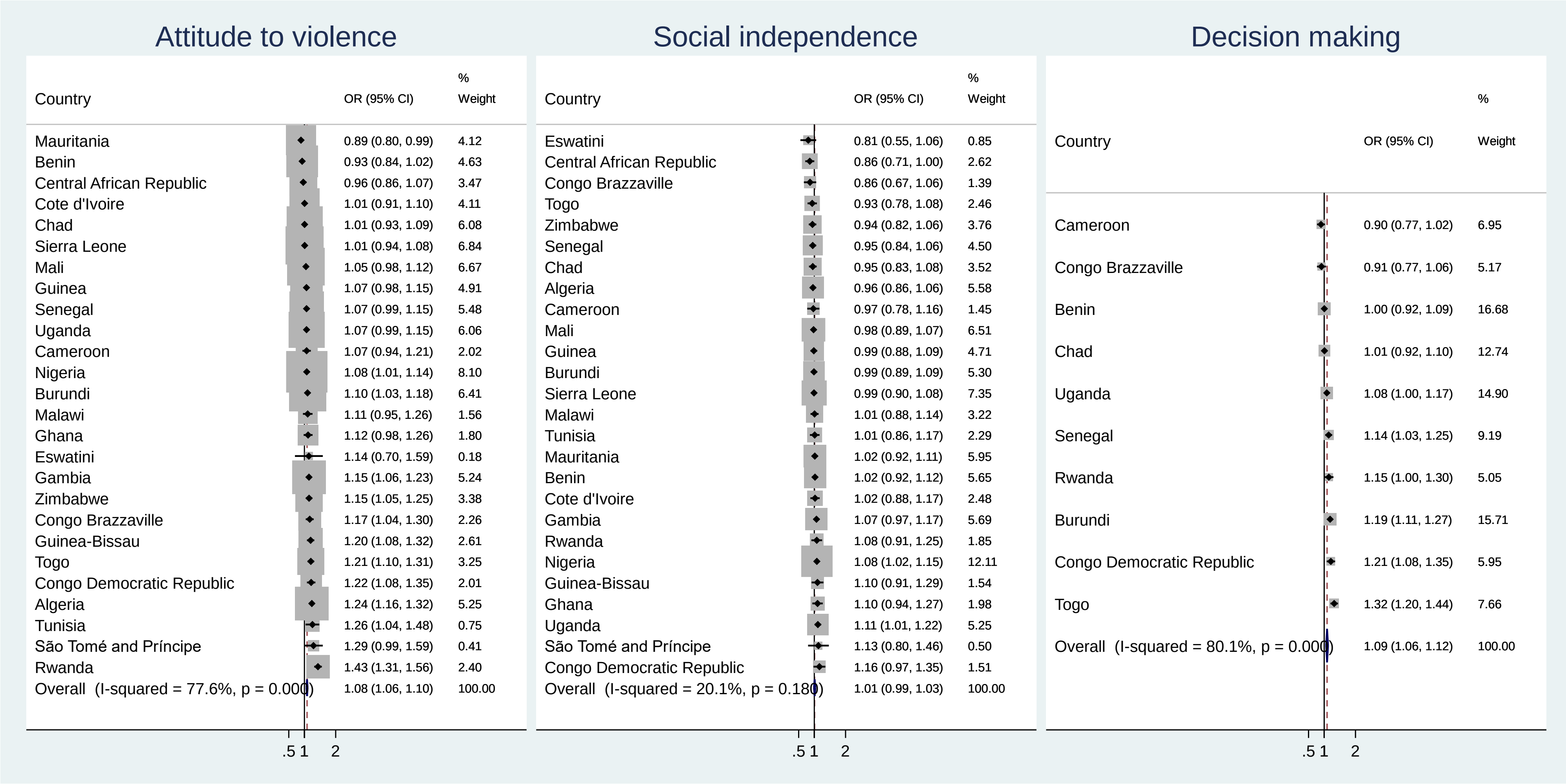

Figure S6. Association between the composite *early childhood development index* and the mother's empowerment level for each SWPER global domain. Coefficients are the crude odds ratios (OR) for a standard deviation increase in the SWPER global score.

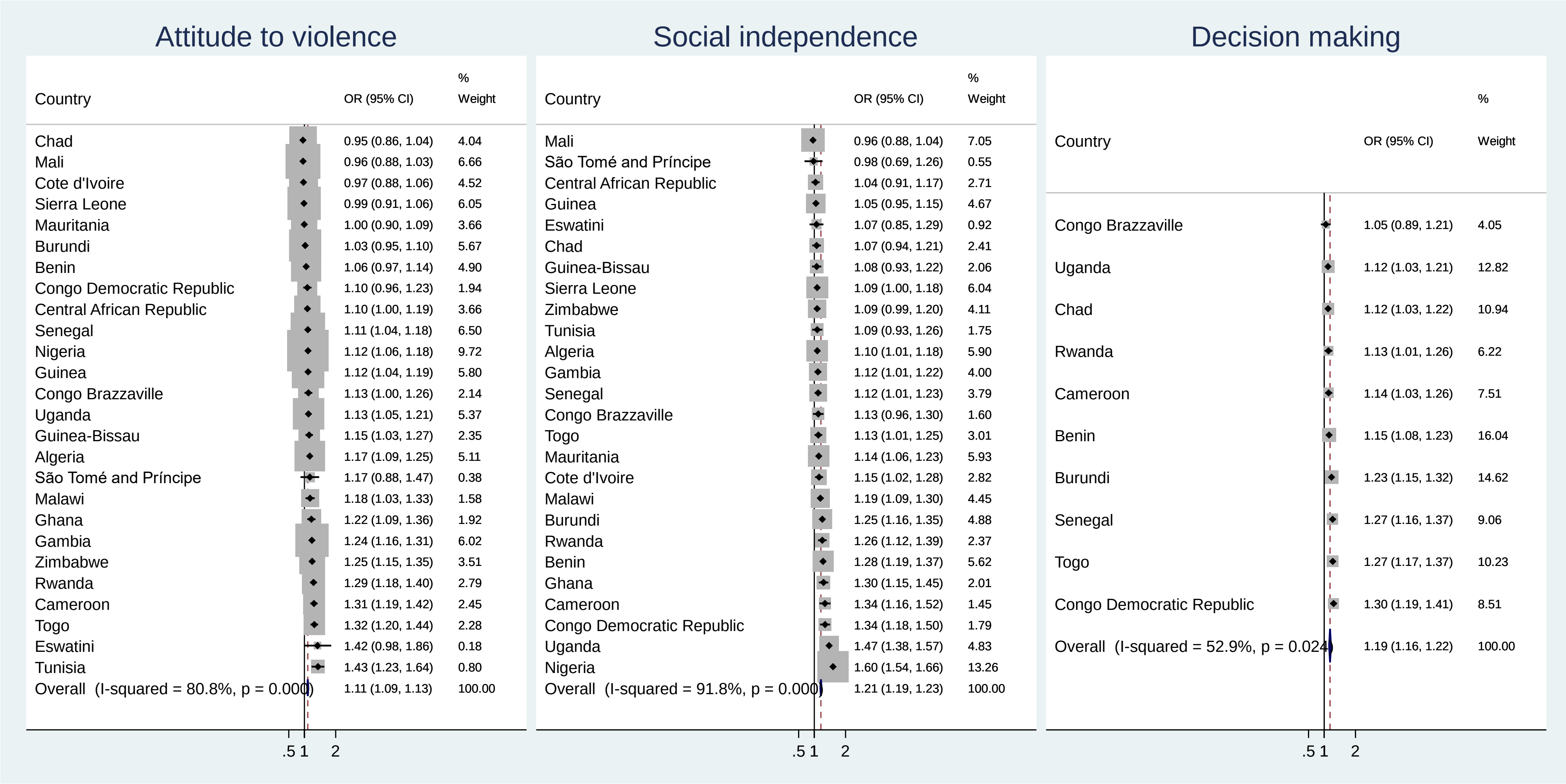

Figure S7. Association between the composite *early childhood development index* and the mother's empowerment level for each SWPER global domain. Coefficients are the odds ratios (OR) for a standard deviation increase in the SWPER global score adjusted for wealth.

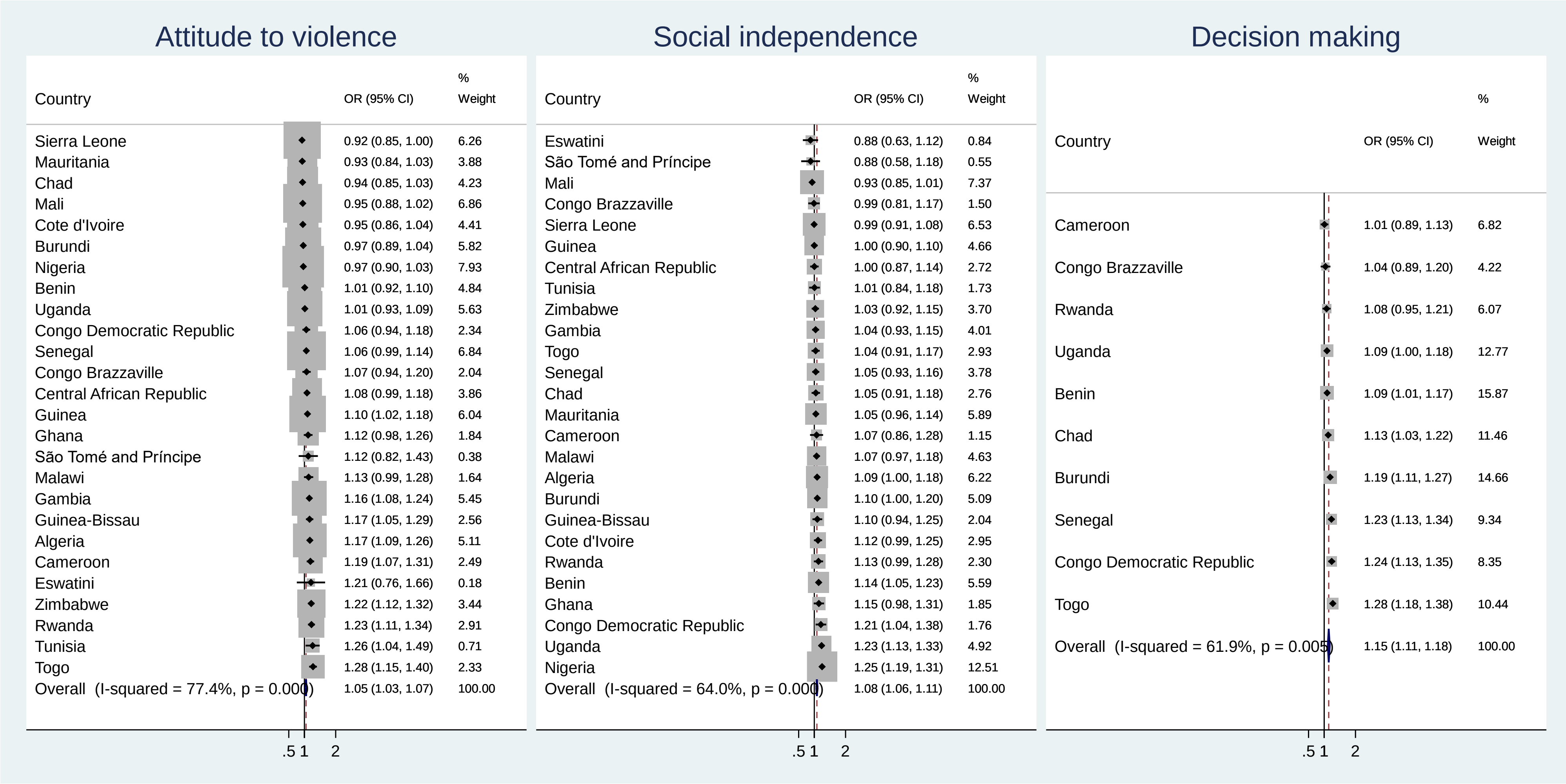

Supplement: Online Supplementary Document [file jogh-10-020406-s001.pdf]
